# Relationship between Age-Dependent Body Constitution and Temporomandibular Joint Sounds in Adolescents

**DOI:** 10.3390/jcm9123927

**Published:** 2020-12-03

**Authors:** Angelika Rauch, Antje Körner, Wieland Kiess, Christian Hirsch, Oliver Schierz

**Affiliations:** 1 Department of Prosthodontics and Materials Science, University of Leipzig, Liebigstr. 12, 04103 Leipzig, Germany; oliver.schierz@medizin.uni-leipzig.de; 2 LIFE—Leipzig Research Center for Civilization Diseases, University of Leipzig, Philipp-Rosenthal-Str. 27, 04103 Leipzig, Germany; antje.koerner@medizin.uni-leipzig.de (A.K.); wieland.kiess@medizin.uni-leipzig.de (W.K.); 3 Department of Women and Child Health, Hospital for Children and Adolescents and Center for Pediatric Research (CPL), University of Leipzig, Liebigstr. 20a, 04103 Leipzig, Germany; 4 Integrated Research and Treatment Center Adiposity Diseases, University of Leipzig, Philipp-Rosenthal-Str. 27, 04103 Leipzig, Germany; 5 Clinic of Pediatric Dentistry, University of Leipzig, Liebigstr. 12, 04103 Leipzig, Germany; christian.hirsch@medizin.uni-leipzig.de

**Keywords:** body constitution, body height, body weight, children, gender, temporomandibular disorders

## Abstract

To date, risk factors for temporomandibular joint (TMJ) sounds are still not completely understood, and anatomical factors are suspected to influence their occurrence. This study aimed to evaluate the impact of body constitution on temporomandibular joint sounds of adolescents. 10- to 18-year-old participants of the LIFE Child Study were examined for TMJ sounds, and physical parameters such as body height, body weight, and general laxity of joints were measured. Odds ratios (OR) for associations of TMJ sounds and standard deviation scores (SDS) of body height and body weight were calculated by using binary logistic regression, including cofactors such as age and number of hypermobile joints. The OR for TMJ sounds and SDS of body height was 1.28 (95% confidence interval (CI) 1.06; 1.56) in females when the age-adjusted height value was above 0. SDS of body weight indicated significant ORs for TMJ sounds in males with values of 0.81 (95% CI 0.70; 0.94). No correlation was detected for SDS values and TMJ crepitus. Tall female adolescents seem to be more prone to TMJ clicking sounds, while their occurrence seems less likely in male adolescents with higher body weight.

## 1. Introduction

Temporomandibular joint (TMJ) sounds are categorized as cardinal symptoms of temporomandibular disorders (TMD) [1]. According to a systematic review, 14.0% of the children or adolescents had clinical TMJ sounds [2], while a current investigation detected an even higher percentage—up to 31.9%—for German adolescents [3]. The gap between prevalence values might be due to the ethnicity of the participants, as previous investigations revealed that anatomical structures, e.g., the angulation of the articular eminence, varies between different ethnical groups [4]. In general, differences in the growth of the maxilla and the mandible in adolescents might lead to an incongruence of the condyle and the articular eminence, and consequently foster TMJ sounds that were, for example, caused by disc displacements (DD) [5,6]. Therefore, the age of young patients seems to be an influencing factor for joint sounds [6]. Moreover, sex [7,8,9] and psychosocial characteristics [10] might be risk factors for TMJ sounds, yet their influence is controversially discussed. A recent investigation observed that psychosocial factors barely influence DD, and it described that some habits like pen biting can act as a risk factor for DD in adolescents [6]. Another reason for TMJ sounds might be the hypermobility of the TMJ. The latter can be induced by a general joint laxity, which is very common in younger patients, and was observed as generalized hypermobility (≥4 joints) in 21.3% of the 10- to 18-year-olds. This mobility usually decreases during pubertal development, however less in girls than in boys, which can be explained by hormonal effects [11]. In most cases, TMJ sounds affect adolescents only minimally, but if painful, the TMJ should be treated, since an impaired oral health-related quality of life and limitations of the jaw movements can occur [12,13]. All in all, the risk factors for TMJ sounds are still not fully understood, and anatomical causes seem to affect changes in the TMJ, which could lead to TMJ sounds. Thus, an influence of the body growth on the onset of TMJ sounds might be possible, and age-dependent tall adolescents might be prone to disc displacement due to rapid growth, whereas a shorter body height might be protective. However, the influence of the body constitution has never been thoroughly investigated.

The present study aims to evaluate the influence of the age-dependent body height and body weight on temporomandibular joint sounds in adolescents. The working hypothesis of this investigation was that the occurrence of TMJ sounds do not depend on the individual body constitutions.

## 2. Material and Methods

A cross-sectional sample of the LIFE Child Study, aged 10 to 18 years, was recruited to investigate physical parameters as well as characteristics of the masticatory and dental system of the participants, the procedure has already been described in detail elsewhere [3,14,15]. Temporomandibular joint sounds were examined by trained dentists who used the examination form of the Diagnostic Criteria for Temporomandibular Disorders (DC/TMD) [16]. This part of the study was approved by the local Ethical Committee (354-10-13122010). For statistical purposes, all reproducible TMJ sounds during vertical and excursive jaw movements were summarized as clinical TMJ sounds [11]. Moreover, the TMJ sounds were separately analyzed, for clicking sounds on the one hand and for crepitus on the other. Physical parameters were examined by calibrated investigators that measured body height, body weight, and general laxity of joints (GJL). According to the reference values for German adolescents, published by Kromeyer-Hauschild et al., standard deviation scores (SDS) were calculated that comprised age- and sex-adapted values for body height and weight [17]. A positive value is comparable to a relatively tall or heavy, and a negative value to a short or thin adolescent, respectively [17]. The Beighton classification was utilized to identify GJL, and it included the examination of nine joints [11,18]. For statistical analysis (SPSS 24, IBM, Armonk, NY, USA), a normal distribution was observed for SDS height values (Shapiro–Wilk test: *p* = 0.132), while age, SDS weight values, and the number of hypermobile joints were not normally distributed (Shapiro–Wilk tests: *p* ≤ 0.001). Consequently, unpaired *t*-tests (normal distribution) and Mann–Whitney U tests (no normal distribution) were utilized, as well as binary logistic regression, to reveal odds ratios (OR) assuming a level of significance of *p* < 0.050. By using binary regression, the odds ratios for TMJ sounds, TMJ clicking, and TMJ clicking in comparison to the age-adjusted height (SDS height) and age-adjusted weight (SDS weight) values were determined, and the potentially confounding effects of hypermobile joints (GJL) were included. The latter were not age-adjusted; thus, age was included as a confounding factor for GJL as well.

## 3. Results

In total, 1116 adolescents (10 to 18 years, 12.9 ± 2.3 years, 51.4% female) were enrolled in the study. For 104 participants, no DC/TMD examination could be performed. For seven participants, no body height was measured, and nine measurements of body weight were not performed.

**Figure 1:**
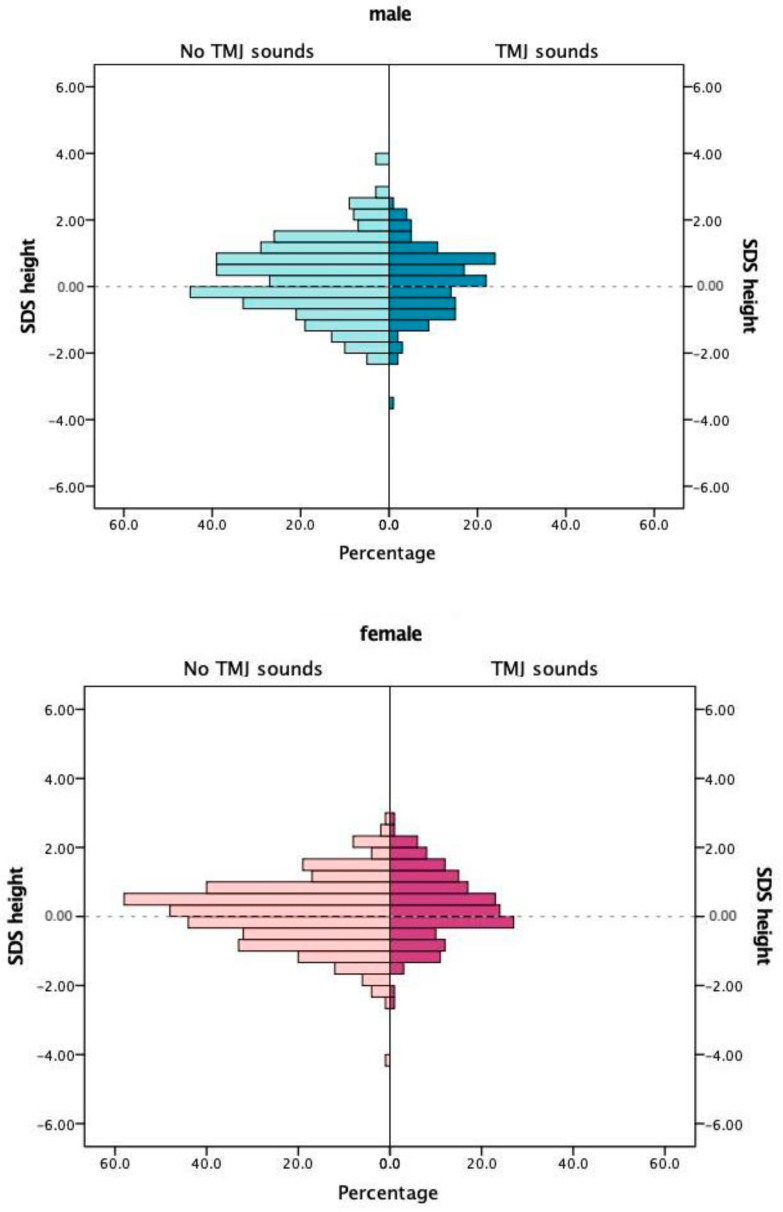
Standard deviation scores (SDS) of body height in relation to temporomandibular joint sounds.

**Figure 2:**
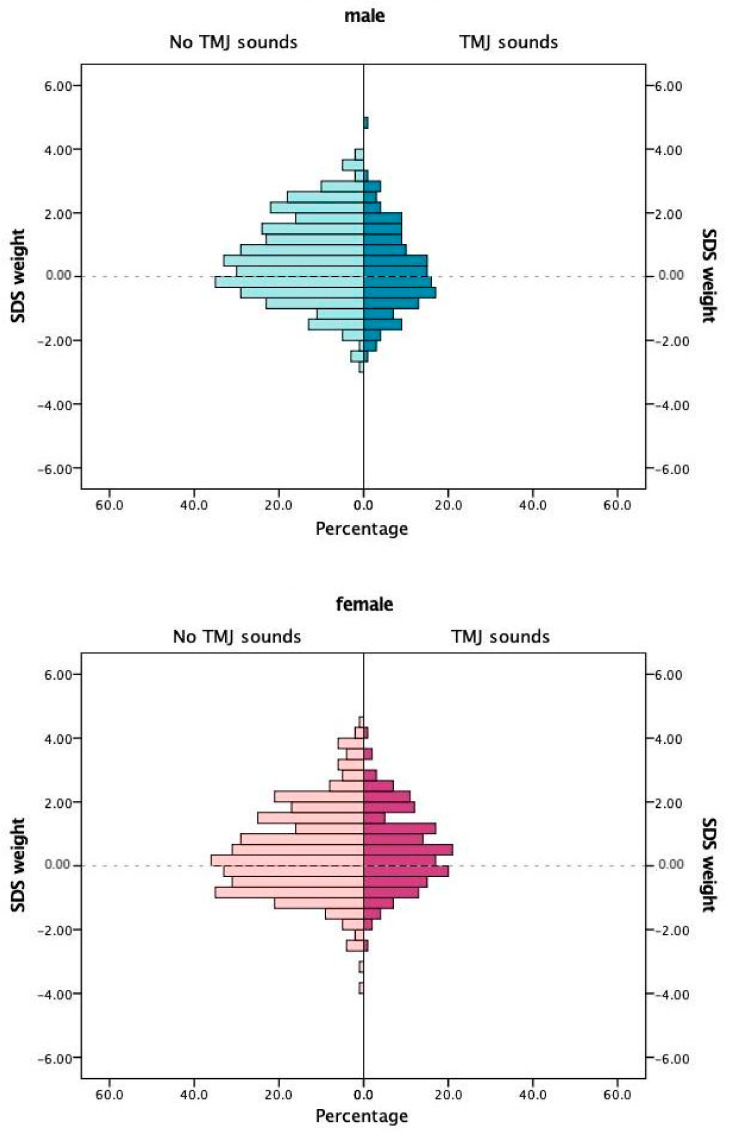
Standard deviation scores (SDS) of body weight in relation to temporomandibular joint sounds.

The comparison of SDS values for body height or body weight of the participants did not yield statistically significant differences for adolescents with or without TMJ sounds (Table 1). A TMJ clicking was observed in 309, and a crepitus in 20 adolescents. Sex-dependent pyramid histograms revealed that TMJ sounds might be correlated to SDS height values in females (Figure 1) and to SDS weight in males (Figure 2).

These results were corroborated by statistical comparisons of SDS height in females that differed significantly (*p* = 0.011) with a mean SDS height of 0.10 without TMJ sounds, and a mean of 0.33 for females with TMJ sounds (Table 2). Moreover, SDS values for body weight in males (0.59/0.24; *p* = 0.006), and the number of hypermobile joints (females), as well as age (females), showed statistically significant differences. Based on these results, four models were created (Table 3) to calculate sex-dependent ORs. When adjusting for cofactors, ORs for SDS height (1.30–1.40, females) and SDS weight (0.75, males) reached significance. Afterwards, TMJ sounds were separately analyzed as click or as crepitus according to the same four models (Table 4 and Table 5). ORs in females revealed values of 1.27–1.38 for clicking sounds and body height, while for body weight of males, ORs of 0.72–0.79 were calculated. For crepitus in both sexes, no statistically significant ORs for SDS height nor SDS weight were detected.

## 4. Discussion

The present investigation aimed to examine the relationship between age- and sex-dependent body constitution and TMJ sounds in adolescents. It was observed that rapid growth in females is a risk factor for TMJ sounds (OR 1.28), which does not apply for crepitus but to clicking sounds revealing an OR of 1.27. TMJ sounds in males do not seem to be affected by being relatively tall but by having age-dependent body weight. Thus, the working hypothesis of this investigation could be partially rejected. Moreover, the results of this study corroborate that age, sex, and general joint laxity influence joint sounds in adolescents.

In most patients, a clicking sound can be explained by the movement of the articular disc onto and off the condyle during jaw movements. This pathologic movement is caused by a displacement of the articular disc (DD) with reduction [19]. The latter is a condition that implicates little or no discomfort for the patient, yet it can induce pain or limitations in jaw movements especially while chewing [6,20]. Previous investigations assumed that a TMJ clicking examined in adolescents can be explained by differences in the growth of the maxilla or the mandible and can be interpreted as a sign of adaption [8]. Even if a treatment is not needed, a careful follow-up is necessary, and patients, as well as their parents, should be informed about the etiology and prognosis of DD [19]. Moreover, it is important to avoid an overtreatment of these adolescents [21]. If necessary, a treatment with reversible/conservative measures, such as manual therapy or splints, can be indicated [22], as it might be useful for adolescents with painful TMJ sounds or DD without reduction with limited opening.

The results of this investigation corroborate the hypothesis that anatomical variations can influence the onset of TMJ clicking sounds in adolescents [6,19]. The authors assume that taller girls can be featured by a low muscle tonus and weakness of the ligaments due to the rapid body growth. Consequently, an abrupt growth spurt in the maxilla or mandible might not be compensated by the anatomical structures. This theory is supported by investigations focusing on body height and other health-related issues in adolescents, as it was observed that body height is associated with knee injuries, especially meniscal injuries, in females [23]. Even lower back pain is positively associated and was determined with an OR of 1.22 [24].

To the surprise of the authors, the results of the present study revealed that body weight seems to be a protective factor to avoid TMJ sounds in male adolescents. The authors assume that higher body weight could be related to more muscular mass or soft tissue, which might obviate potential disc displacements in adolescents.

In the present study, some data could not be collected, which can be explained by the fact that some participants/parents rejected parts of the examination. This might be due to the high number of questionnaires and examinations, which could have been exhausting for the adolescents and could have led to a lower adherence. Moreover, organizational reasons are possible, since dental and physical examinations were not performed on the same day. This investigation only included the examination of German adolescents, yet it is reported that ethnical anatomical variances are possible in adolescents [4]. Therefore, the measured values for height and weight were transformed to SDS values with the help of normative data from the German adolescent population. Thus, the SDS values can be easily compared to SDS values observed in other countries. Another limiting factor is that joint sounds were only clinically examined. However, with regard to the costs and estimated time of methods to verify the results like ultrasonography or magnetic resonance [19], the clinical examination was preferred. Moreover, results for TMJ crepitus should be interpreted with caution, since it occurred only in a few adolescents.

The strengths of this study are the large sample size of approximately 1000 participants, which reduces the risk for confounding and bias. Moreover, standardized examination methods were used by trained investigators. The results of the current study provide additional information on the etiology of TMJ sounds in adolescents and describe their relationship to the body constitution of adolescents for the first time. To corroborate results of this investigation, future longitudinal research projects should be created to verify if rapid growth over a distinct period of time influences TMJ sounds in a multinational approach.

The results of this investigation can help to improve healthcare and management of TMD in adolescents, since dentists, orthodontists, and pediatricians should be aware that taller girls seem to be more prone to DD with reduction. Further research on the etiology of disc displacements in adolescents will help to fully understand the influencing factors of TMJ sounds.

## 5. Conclusions

TMJ sounds in adolescents are influenced by sex, body constitution, and general joint laxity. Tall female adolescents are more prone to TMJ clicking sounds. Increased age-dependent body weight seems to be a protective factor to prevent the onset of TMJ clicking sounds in male adolescents.

## Figures and Tables

**Table 1:** Means and standard deviation (SD) of physical variables in relation to temporomandibular joint (TMJ) sounds (both sexes); SDS: standard deviation scores, CI: confidence interval, n.s.: not significant.

Parameter	TMJ Sound—Negative				TMJ Sound—Positive				95% CI of Difference	*p*-Value
	*n*	Mean (SD)	Min	Max	*n*	Mean (SD)	Min	Max		
SDS height	686	0.18 (1.05)	−4.01	3.87	322	0.25 (0.98)	−3.41	2.71	−0.20; 0.07	n.s. ^†^
SDS weight	684	0.53 (1.36)	−3.79	4.49	322	0.40 (1.24)	−2.52	4.78	−0.05; 0.30	n.s. ^‡^
Number of hypermobile joints	683	1.82 (1.82)	0	7	319	2.09 (1.90)	0	7	−0.51; −0.02	0.032 ^‡^
Age in years	689	13.0 (2.2)	10	18	323	13.4 (2.3)	10	18	−0.63; −0.06	0.019 ^‡^

^†^ unpaired *t*-test, ^‡^ Mann–Whitney U test.

**Table 2:** Sex-dependent means and standard deviation (SD) of physical variables in relation to TMJ sounds; SDS: standard deviation scores, CI: confidence interval, n.s.: not significant.

Parameter	TMJ Sounds—Negative		TMJ Sounds—Positive		95% CI of Difference	*p*-Value
	*n*	Mean (SD)	*n*	Mean (SD)		
Male						
SDS height	336	0.27 (1.13)	150	0.15 (0.99)	−0.08; 0.32	n.s. ^†^
SDS weight	335	0.59 (1.31)	150	0.24 (1.28)	0.10; 0.60	0.006 ^‡^
Number of hypermobile joints	333	1.53 (1.64)	149	1.63 (1.64)	−0.42; 0.21	n.s. ^‡^
Age in years	337	13.0 (2.1)	151	13.0 (2.1)	−0.47; 0.34	n.s. ^‡^
Female						
SDS height	350	0.10 (0.96)	172	0.33 (0.97)	−0.40; −0.05	0.011 ^†^
SDS weight	349	0.50 (1.40)	172	0.55 (1.19)	−0.31; 0.16	n.s. ^‡^
Number of hypermobile joints	350	2.11 (1.92)	170	2.49 (2.02)	−0.75; −0.02	0.040 ^‡^
Age in years	352	13.1 (2.2)	172	13.7 (2.3)	−0.99; −0.18	0.005 ^‡^

^†^ unpaired *t*-test, ^‡^ Mann-Whitney U test.

**Table 3:** Multivariable analyses of TMJ sounds (N = 322) in relation to physical variables; SDS: standard deviation scores, OR: odds ratio, CI: confidence interval, n.s.: not significant.

TMJ Sounds		Male			Female		
Model	Variables	OR	95% CI	*p*-Value	OR	95% CI	*p*-Value
1a	SDS height	0.90	0.75; 1.08	n.s.	1.28	1.06; 1.56	0.013
1b	SDS weight	0.81	0.70; 0.94	0.007	1.04	0.91; 1.20	n.s.
2	SDS height	1.13	0.89; 1.45	n.s.	1.33	1.06; 1.65	0.011
	SDS weight	0.75	0.61; 0.93	0.007	0.95	0.81; 1.11	n.s.
3	SDS height	1.13	0.88; 1.45	n.s.	1.30	1.04; 1.62	0.023
	SDS weight	0.75	0.61; 0.93	0.009	0.98	0.83; 1.15	n.s.
	Number of hypermobile joints	1.00	0.89; 1.13	n.s.	1.10	1.00; 1.21	n.s.
4	SDS height	1.14	0.89; 1.46	n.s.	1.40	1.13; 1.76	0.004
	SDS weight	0.75	0.61; 0.93	0.008	0.95	0.81; 1.12	n.s.
	Number of hypermobile joints	1.01	0.89; 1.14	n.s.	1.09	0.99; 1.20	n.s.
	Age	1.02	0.93; 1.12	n.s	1.16	1.06; 1.26	0.001

**Table 4:** Multivariable analyses of TMJ clicking (N = 309) in relation to physical variables; SDS: standard deviation scores, OR: odds ratio, CI: confidence interval, n.s.: not significant.

TMJ Clicking		Male			Female		
Model	Variables	OR	95% CI	*p*-Value	OR	95% CI	*p*-Value
1a	SDS height	0.89	0.74; 1.06	n.s.	1.27	1.05; 1.55	0.016
1b	SDS weight	0.79	0.67; 0.92	0.003	1.04	0.91; 1.20	n.s.
2	SDS height	1.15	0.89; 1.47	n.s.	1.32	1.06; 1.64	0.015
	SDS weight	0.72	0.58; 0.90	0.003	0.95	0.81; 1.11	n.s.
3	SDS height	1.15	0.89; 1.48	n.s.	1.28	1.02; 1.60	0.031
	SDS weight	0.72	0.58; 0.90	0.003	0.98	0.84; 1.16	n.s.
	Number of hypermobile joints	0.98	0.87; 1.11	n.s.	1.11	1.01; 1.22	0.033
4	SDS height	1.15	0.89; 1.48	n.s.	1.38	1.10; 1.74	0.006
	SDS weight	0.72	0.58; 0.90	0.003	0.96	0.82; 1.13	n.s.
	Number of hypermobile joints	0.98	0.87; 1.11	n.s.	1.11	1.00; 1.22	0.046
	Age	1.00	0.90; 1.10	n.s.	1.16	1.06; 1.26	0.001

**Table 5:** Multivariable analyses of TMJ crepitus (N = 20) in relation to physical variables; SDS: standard deviation scores, OR: odds ratio, CI: confidence interval, n.s.: not significant.

TMJ Crepitus		Male			Female		
Model	Variables	OR	95% CI	*p*-Value	OR	95% CI	*p*-Value
1a	SDS height	1.00	0.60; 1.66	n.s.	1.04	0.48; 2.26	n.s.
1b	SDS weight	1.08	0.71; 1.64	n.s.	0.91	0.52; 1.62	n.s.
2	SDS height	0.91	0.47; 1.75	n.s.	1.15	0.47; 2.78	n.s.
	SDS weight	1.13	0.67; 1.91	n.s.	0.87	0.45; 1.69	n.s.
3	SDS height	0.85	0.45; 1.64	n.s.	1.15	0.47; 2.80	n.s.
	SDS weight	1.26	0.74; 2.17	n.s.	0.86	0.44; 1.71	n.s.
	Number of hypermobile joints	1.35	1.00; 1.81	n.s.	1.00	0.67; 1.47	n.s.
4	SDS height	0.97	0.51; 1.84	n.s.	1.23	0.50; 3.01	n.s.
	SDS weight	1.18	0.70; 1.99	n.s.	0.85	0.44; 1.66	n.s.
	Number of hypermobile joints	1.37	1.02; 1.84	0.037	0.99	0.67; 1.47	n.s.
	Age	1.20	0.93; 1.55	n.s.	1.17	0.84; 1.62	n.s.

## Data Availability

Data available on request from the corresponding author due to restrictions, e.g., privacy or ethical reasons.
